# Aerosol Jet Printing of Silver Lines with A High Aspect Ratio on A Heated Silicon Substrate

**DOI:** 10.3390/ma13030730

**Published:** 2020-02-05

**Authors:** Alexey Efimov, Pavel Arsenov, Denis Kornyushin, Anna Lizunova, Ivan Volkov, Victor Ivanov

**Affiliations:** Moscow Institute of Physics and Technology (National Research University), Dolgoprudny 141701, Russia; kornush94@rambler.ru (D.K.); anna.lizunova@gmail.com (A.L.); volkov256@yandex.ru (I.V.); ivanov.vv@mipt.ru (V.I.)

**Keywords:** aerosol nanoparticles, printed electronics, aspect ratio, microstructure, sintering, silver, electrical resistivity, 3D manufacturing

## Abstract

In this work, we studied the formation of conductive silver lines with high aspect ratios (*AR* = thickness/width) > 0.1 using the modernized method of aerosol jet printing on a heated silicon substrate. The geometric (*AR*) and electrical (resistivity) parameters of the formed lines were investigated depending on the number of printing layers (1–10 layers) and the temperature of the substrate (25–300 °C). The *AR* of the lines increased as the number of printing layers and the temperature of the substrate increased. An increase in the *AR* of the lines with increasing substrate temperature was associated with a decrease in the ink spreading as a result of an increase in the rate of evaporation of nano-ink. Moreover, with an increase in the substrate temperature of more than 200 °C, a significant increase in the porosity of the formed lines was observed, and as a result, the electrical resistivity of the lines increased significantly. Taking into account the revealed regularities, it was demonstrated that the formation of silver lines with a high *AR* > 0.1 and a low electrical resistivity of 2–3 μΩ∙cm is advisable to be carried out at a substrate temperature of about 100 °C. The adhesion strength of silver films formed on a heated silicon substrate is 2.8 ± 0.9 N/mm^2^, which further confirms the suitability of the investigated method of aerosol jet printing for electronic applications.

## 1. Introduction

Currently, there is great interest in the manufacture of low-cost electronic devices using printing technologies [1,2]. The use of these technologies allows the formation of active and passive components by direct deposition of functional materials in the form of nanoparticles on the surface of the substrate without the use of expensive photolithographic masks and stencils [3,4,5,6]. Among the existing printing methods, aerosol jet printing is the most promising technology because it allows the formation of functional elements with a minimum lateral size of up to 10 microns [7,8,9] both on flat and curved substrates [10,11]. This technology has been applied for the manufacture of transistors [12], sensors [13,14,15], solar cells [16], microheaters [17], microantennas [18,19] and interconnects [20]. Previous studies of this technology relied mainly on the formation of thin-film microstructures due to the need for the manufacture of planar electronic devices. At the same time, there is a large and inevitable production demand for the manufacture of narrow and abnormally high bulk microstructures having aspect ratios (ratio of thickness to line width) of more than 0.1. These microstructures, for example, are relevant for use as busbars and fingers of solar cells, where minimization of dimming losses due to narrow line widths and minimization of power losses due to high line thicknesses are required [21].

There are not many studies reporting the study of ways to increase the aspect ratio of lines formed by aerosol jet printing. Therefore, earlier in the work [22], it was established that in a single-pass mode of aerosol printing due to the optimization of rates of aerosol and sheath gas, it is possible to obtain narrow (20 μm) lines with an aspect ratio of more than 0.1. At the same time, it was shown in [23], that an increase in the number of print passes and a decrease in the speed of movement of the substrate do not lead to an increase in the aspect ratio of the lines due to the effect of ink spreading during aerosol jet printing. Obtaining lines with a high aspect ratio is possible only using additional approaches for processing the substrate or the deposited material. Therefore, one of the approaches is to structure the substrate using imprinting or lithography, which limits the spreading of ink on it [24], and the second approach is based on the use of multiple printing passes in combination with local laser sintering of the deposited material [25,26,27,28]. Both presented approaches significantly complicate and increase the cost of the aerosol jet printing process through the use of additional lithographic, laser and optomechanical equipment.

In this regard, there is still a need to develop and study more technologically simple methods that ensure the formation of lines with a high aspect ratio. Therefore, in this paper, we proposed to investigate a fairly simple method of aerosol jet printing of lines on a thermally heated substrate, in which the conditions for forming lines with a high aspect ratio will be ensured due to the temperature effect on the deposited material and many printing passes. In addition, in this work, a comparative study of the geometric parameters, structure, morphology, and electrical resistivity of lines of nanoparticles deposited on an unheated and heated substrate with the potential of their use as current-carrying compounds will be performed.

## 2. Experimental 

**Figure 1:**
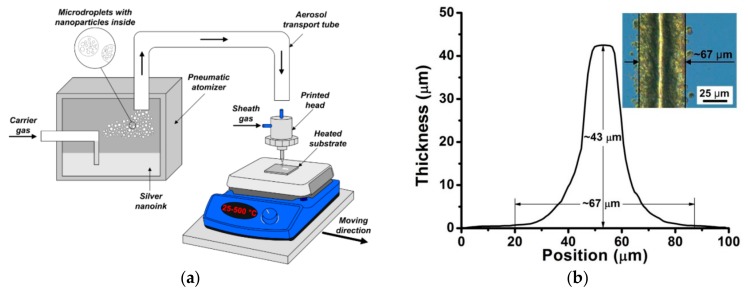
Schematic of an experimental setup for focused deposition of aerosol particles on a heated silicon substrate (**a**); An example of a cross-sectional profile of a silver line, measured with an optical profilometer, where the measured thickness t, width W and aspect ratio *AR* of the line are ~ 67 μm, ~ 43 μm and 0.64, respectively. The characteristic lengths of the formed lines in the experiments ranged from 10 to 30 mm. The inset shows the optical image of this line (**b**).

In the experiments, the formation of the studied lines was carried out on a modernized commercial aerosol jet printer AJ 15XE (Neotech AMT GmbH, Nuremberg, Germany). The modernization of the aerosol jet printer was to provide controlled heating of the substrate in the process of aerosol printing using heating plate in the temperature range from 25 to 500 °C. The experimental setup, including a modernized aerosol jet printer for the deposition of silver lines on a heated silicon substrate, is shown in Figure 1.

Aerosol particles in the form of micro droplets with nanoparticles inside were generated using a pneumatic atomizer during spraying a mixture of silver nano-ink PG-007 (Paru Co. Ltd., Seoul, South Korea) based on solvents 1-methoxy-2-propanol (MOP) and ethylene glycol (EG) in a volume ratio of 3:1, respectively. The sheet resistivity of the sintered nano-ink layer was 3.5 mΩ/sq according to the manufacturer's specification [29]. The mass concentration of silver nanoparticles in the ink was about 46%. Nanoparticles had a size in the range from 5 to 420 nm with a mean size of 108 ± 68 nm according to the results of measurements by transmission electron microscope JEM-2100 (JEOL Ltd., Tokyo, Japan). Pure nitrogen N2 (99.9999%) was used as the carrier gas. Next, the aerosol particles were transport to a print head with a micro nozzle for focusing and deposition on a moving heated silicon substrate. In the experiments, a micro nozzle with an outlet diameter of 150 μm was used, and the distance from the micro nozzle to the substrate was 5 mm. The line was deposited when the substrate was moved relative to the nozzle with a constant printing speed of the order of *V*_s_ = 100 mm/min. The characteristic lengths of the formed lines in the experiments ranged from 10 to 30 mm. The control of the line width *W* was carried out by changing the values of the focusing ratio (*FR* = *Q*_sh_/*Q*_a_) with varying flow rates of aerosol *Q*_a_ and constant sheath gas *Q*_sh_ = 90 sccm. The possibility of increasing the thickness of the formed lines was achieved due to several layers of printing on each other in the multi-pass mode. In this case, the temperature of the substrate was a critical parameter, since it affected the spreading of nano-ink due to a change in the drying rate, evaporation, and solidification of the nano-ink.

Experiments to determine the conditions and patterns of silver line formation with a high aspect ratio and low resistivity were performed in two stages. At the first stage, a study was made of the temperature ranges responsible for the drying and sintering of the deposited silver nano-ink in order to obtain monolithic lines with a low resistivity. To do this, we studied the dependence of the electrical resistivity of silver lines on heat treatment of nano-ink in the range from 25 to 500 °C, and also established the nature of the dependence of the measured resistance on temperature in the range from 25 to 100 °C for sintered and dried lines. Sintered lines were those that were dried and sintered, and dried lines were those that had only been dried. In this case, the drying process involved the removal of the solvent and surfactants included in the composition of the used nano-ink, and the sintering process was responsible for monolithization of the deposited nano-ink.

At the second stage, we studied the effect of the number of printing layers *N* (from 1 to 10) and the temperature of the heated substrate *T*_s_ (from 25 to 300 °C) on the values of width *W*, thickness *t*, aspect ratio *AR* and electrical resistivity *ρ*, as well as structural and morphological features of the formed lines. The values of the variable and fixed parameters of the aerosol jet printing process used in the experiments are presented in Table 1. In the process of aerosol jet printing on a heated substrate in several layers, each subsequent layer was deposited immediately after printing of the previous layer without additional exposure to time. In this case, experiments on printing lines on a heated substrate were carried out with completely heated substrate. The total heating time of the substrate was from 10 to 20 min, depending on the set temperature. After the process of aerosol jet printing, the obtained lines were additionally sintered on a heating plate at a temperature of 400 °C for 60 minutes in order to ensure their conductive properties.

In order to demonstrate the effect of ink spreading on the substrate the *FR* = *Q*_sh_/*Q*_a_ was reduced from 5 to 2 in experiments to establish the effect of the *T*_s_ (25, 50, 100, 200 and 300 °C) on the aspect ratio of lines (see Table 1). The value of the *FR* was reduced due to an increase in the amount of aerosol *Q*_a_ deposited on the substrate at *Q*_sh_ = const. Moreover, earlier in [22] it was already shown that increasing the value of the *FR* allows increasing the values of the aspect ratio of the printed lines.

The aspect ratio (*AR*) of the lines was determined from the analysis of the cross-sectional profile of the line in accordance with Equation (1):

(1) $$AR=\frac{t}{W},$$

where *t* – line thickness, *W* – line width.

The line cross-sectional profile was measured using a DCM 3D optical profilometer (Leica Microsystems GmbH, Wetzlar, Germany). This profilometer allows to obtain an averaged cross-sectional profile of lines. The profile was averaged over 50 lines section with an analysis area of 200 × 200 μm^2^. As an example, Figure 1b shows the measured line cross-sectional profile for which a thickness *t* ~ 43 μm, a width *W* ~ 67 μm, and an aspect ratio of the line *AR* ~ 0.64 were determined. The electrical resistivity of the lines *ρ* was determined using Equation (2):

(2) $$\rho =R\frac{S}{L},$$

where *R* – line resistance, *S* – line cross-sectional area, *L* – line length.

The line resistance *R* was measured using the four-point probe method [30]. For this purpose, four contacts were formed on the surfaces of the silver lines using conductive silver paste PELCO® # 16031 (Ted Pella, Inc., Redding, CA, USA). A current *I* in the range of 10 to 100 μA was passed through two external contacts using a current source SourceMeter 2401 (Tektronix, Inc., Beaverton, OR, USA). The voltage drop *V* between two internal contacts was measured using a U1253B multimeter (Agilent Technologies, Inc, Santa Clara, CA, USA). Based on the measurement data, *I* was obtained as a function of *V*, which had a linear character and from which the resistance of printed lines *R* was calculated as the slope of *I*(*V*) plot. The cross-sectional area of line *S* was measured using an optical profilometer DCM 3D (Leica Microsystems GmbH, Wetzlar, Germany), and the line length *L* was measured using an optical microscope VHX-1000 (KEYENCE Corp. of America, Itasca, IL, USA). The structural and morphological features of the lines were determined using a JSM 7001F scanning electron microscope (JEOL, Ltd., Tokyo, Japan).

In order to confirm the suitability of using a thermally heated substrate in the process of aerosol jet printing, we measured the adhesion strength of silver films with an area of 15x15 mm^2^ formed on a heated silicon substrate at *T*_s_ = 100 °C, *N* = 5 layers, *FR* = 2, and *V*_s_ = 1000 mm/min and sintered at 400 °C for 60 minutes. The adhesion measurements were carried out using a pull-off adhesion analyzer LUMiFrac (LUM GmbH, Berlin, Germany) according to ISO 4624 [31]. The glue Super Moment (Henkel Rus, Ltd., Moscow, Russia) was used as an adhesive between printed layer and pull off test stamp with bonded area of 78.5 mm^2^. The glued parts were cured at room temperature for 12 hours according to the manufacturer's datasheet. The samples of this study have been tested with a linear load increase of 5 N/s and a temperature of 25 °C. Fourteen samples were analyzed in pull off test. The tensile strength σ [32] are calculated using the centrifugal force *F*_c_ and the bonded area *A* by the following Equation (3):

(3) σ=Fc/A,

Thus, based on the results of the studies, regularities for the formation of lines on a heated substrate were established and conditions that ensure the production of lines with a high aspect ratio and minimum electrical resistivity were found.

## 3. Results and Discussion

Lines with high aspect ratios (*AR* = thickness/width) are required for many applications in printed electronics [22]. In addition, the lines must have a low resistivity required to minimize resistive losses in manufactured electronic devices [33,34]. The low resistivity of the lines of nanoparticles is ensured when the nanoparticles are continuously connected to each other, and the bulk structure of the line has a low porosity [35]. As a rule, this is achieved by thermal drying and subsequent sintering of the deposited lines, provided that the nanoparticle structure is densely formed [35,36]. Therefore, in the drying process at relatively low temperatures < 150 °C, the solvent and surfactants are removed from the surface of the nanoparticles, and in the process of sintering of the nanoparticles at high temperatures > 250 °C, the formation of many contacts between nanoparticles takes place, and a bulk monolitic structure is formed [37].

In aerosol jet printing of lines on a heated substrate, depending on its temperature *T*_s_, the processes of deposition, spreading, drying, and sintering of nanoparticles can occur in parallel. For this reason, the substrate temperature *T*_s_ can significantly affect both the aspect ratio *AR* of the formed lines due to the influence on the spreading, drying and evaporation rates of the nano-ink, and their morphology, structure and deposition density of nanoparticles, and, as a consequence, on the electrical resistivity of the lines. Considering this, before the experiments on the deposition of lines on a heated substrate, the study investigated the effect of heat treatment used nano-ink on resistivity *ρ*, conductivity, structure and morphology of the lines in order to determine the temperature ranges responsible for the drying and sintering processes.

**Figure 2:**
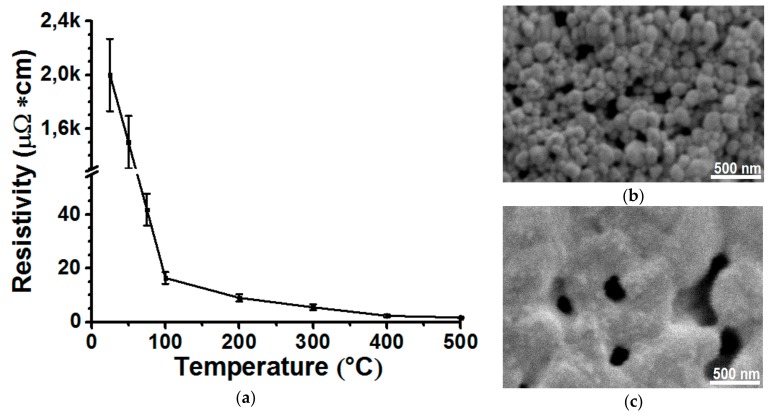
Dependence of the electrical resistivity of the lines from silver nano-ink on the temperature of the substrate heating (**a**); SEM image of a line dried at 100 °C (**b**) and sintered at 400 °C (**c**), respectively. The formation of the lines was carried out at *T*_s_ = 25 °C, *N* = 1 layer, *FR* = 5 and *V*_s_ = 100 mm/min.

Figure 2a shows the temperature dependence of the electrical resistivity *ρ* of the line of silver nano-ink in the range from 25 to 500 ° C during the heat treatment. The error bars shown in Figure 2a are the expanded uncertainty determined from the measurements of 10 lines with a 95% confidence probability [38]. The formation of this line was carried out at *T*_s_ = 25 °C, *N* = 1 layer, *FR* = 5 and *V*_s_ = 100 mm/min. It is shown from Figure 2 that the electrical resistivity ρ of the initial thermally untreated line at 25 °C is extremely high and amounts to ~ 2000 μΩ∙cm. In this case, a significant drop in the line electrical resistivity by two orders of magnitude from ~ 2000 to ~ 16 μΩ∙cm is achieved with increasing temperature from 25 to 100 °C, respectively. At a temperature of about 100 °C, the drying process of the line is probably completed as a result of evaporation of the main fraction of the solvent mixture (1-methoxy-2-propanol and ethylene glycol) and decomposition of the organic surfactants included in nano-ink. Confirmation of this statement is presented in Figure 2b, where the SEM image of the surface of the line heat-treated at 100 °C shows that the line surface has a structure consisting of many individual micro-sized agglomerates of nanoparticles weakly bonded to each other, which is typical for dried structures from nanoparticles.

Figure 2a also shows that with a further increase in the heating temperature from 100 to 300 °C, the electrical resistivity of the line changes less significantly from ~ 16 to ~ 6 μΩ∙cm, respectively. This temperature range is probably responsible for the beginning of the stage of the sintering process of nanoparticles, at which contacts and necks between nanoparticles begin to form. Moreover, it can also be seen from the graph in Figure 2a that the complete sintering of nanoparticles occurs in the temperature range from 300 to 500 °C, where the line electrical resistivity reaches minimum values of the order of ~ 2.3 μΩ∙cm and then ceases to change with increasing substrate temperature [39,40]. In the SEM image of the surface of the line heat-treated at 400 °C, it can be seen that the line consists of a bulk monolitized material with a certain number of micro-sized pores formed during sintering of nanoparticles. These structural features and line morphology are characteristic of sintered nanoparticle structures, where the presence of pores in the line structure also causes a nearly 2-fold difference between the electrical resistivity of the sintered silver line and the bulk silver resistivity of 1.6 μΩ∙cm.

**Figure 3:**
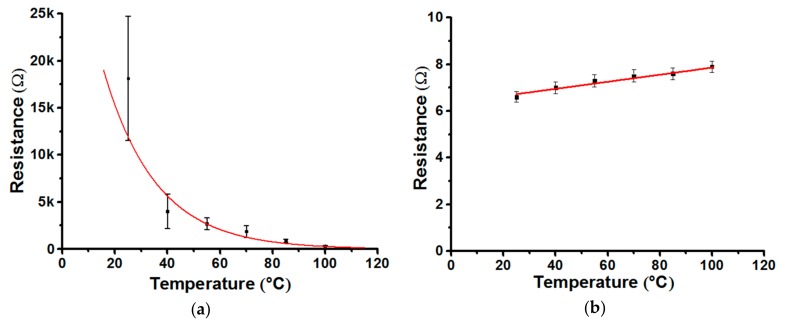
Temperature dependence of the resistance of dried and sintered lines after heat treatment for 60 minutes formed at temperatures of 100 °C (**a**) and 400 °C (**b**), respectively. The formation of this lines was carried out at *T*_s_ = 25 °C, *N* = 1 layer, *FR*=5 and *V*_s_ = 100 mm/min.

Thus, by analyzing the dependence of the electrical resistivity of the line of nanoparticles on the heating temperature of the substrate (Figure 2a), it was found that the drying and sintering processes used by the nanoparticles are completed at temperatures of approximately 100 °C and 400 °C, respectively. An additional confirmation of this statement is the results of measurements of the nature of the temperature dependence of the heat-treated lines at 100 °C and 400 °C for 60 min, respectively. In Figure 3, the samples dried at 100 °C and sintered at 400 °C demonstrate a different character of the dependence of resistance on temperature.

The error bars shown in Figure 3a,b are the expanded uncertainty determined from the measurements of ten lines with a 95% confidence probability. The resistance of the dried samples has a semiconductor nature of conductivity, since it decreases exponentially with increasing temperature, see Figure 3a. Figure 3a shows that with increasing line temperature from 25 to 100 °C, the resistance of the dried lines decreases from 18 to 0.3 kΩ, respectively. In this case, the resistance of sintered lines increases linearly from 6.6 to 8.0 Ω with an increase in the line temperature from 25 to 100 °C (Figure 3b), which is a characteristic for metal structures.

Subsequently, the established temperature ranges responsible for the drying and sintering of particles were used in experiments of aerosol jet printing on a heated substrate in order to obtain lines with a high aspect ratio and low electrical resistivity.

**Figure 4:**
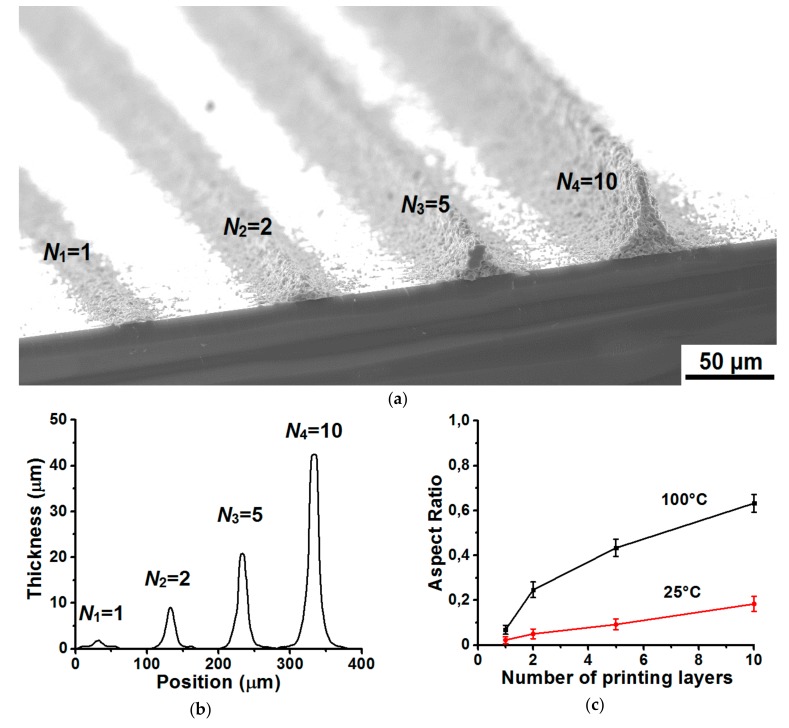
SEM image of silver lines formed by aerosol jet printing on heated silicon substrate at *T*_s_ = 100 °C with a change in the number of printing layers *N* from 1 to 10, where *V*_s_ = 100 mm/min and *FR* = 5 (**a**) and their relevant cross-sectional profiles measured on an optical profilometer (**b**). The dependence of the values of the aspect ratio of lines on the number of printing layers *N* is from 1 to 10 when printing on heated substrate at *T*_s_ = 100 °C and on unheated substrate at *T*_s_ = 25 °C (**c**).

Figure 4a shows SEM images of silver lines formed by aerosol jet printing on a heated silicon substrate at *T*_s_ = 100 °C, *V*_s_ = 100 mm/min and *FR* = 5, depending on the number of printing layers *N* = 1, 2, 5, and 10. The lines after printing were additionally sintered on a heating plate at a temperature of 400 °C for 60 minutes in order to ensure their conductive properties. Figure 4b shows their respective cross-sectional profiles. Figure 4a,b show that with an increase in the number of printing layers *N* from 1 to 10 when particles are deposited on a heated substrate at *T*_s_ = 100 °C, the thickness of the lines t increases from ~ 2 to ~ 43 μm with a change in their width *W* from ~ 37 to ~ 66 μm, respectively. This increase is probably due to the fact that when printing on a heated substrate during the deposition of ink material on a heated surface, the ink dries quickly, without having time to spread on the surface, and the subsequent layer of ink material is deposited already on the annealed line.

From the analysis of the cross-sectional profiles of the lines shown in Figure 4b, the aspect ratio *AR* of the lines was determined and their dependence on the number of printing layers *N* (1–10) was plotted when printing on a heated substrate at *T*_s_ = 100 °C, see Figure 4c. A similar analysis was also performed for lines formed on an unheated substrate at *T*_s_ = 25 °C. Figure 4c shows that the aspect ratio of the lines formed on the heated substrate at *T*_s_ = 100 °C almost linearly increases with the number of printing layers *N*. For example, *AR* values increase from 0.06 to 0.63 with an increase in the number of printing layers *N* is from 1 to 10, respectively. In this case, the electrical resistivity ρ of the sintered lines is ~ 2.4 μΩ∙cm.

In case of printing on unheated substrate at *T*_s_ = 25 °C, the absolute values of the aspect ratio *AR* of lines reach lower values in comparison with the printing mode on heated substrate at *T*_s_ = 100 °C. For example, the aspect ratio of the lines is 0.18 and 0.63 at a temperature of the heated substrate of 25 °C and 100 °C, respectively. This indicates that during the deposition of lines on unheated substrate, due to a greater spreading, the nano-ink along with an increase in the line thickness *t* due to a larger amount of deposited material substantially increases its width *W* in accordance with the expression *AR* = (thickness/width). Thus, it was experimentally found that using a heated substrate *T*_s_ = 100 °C and a multitude of printing layers, it is possible to multiply increase the aspect ratio of lines in proportion to the number of layers *N*.

However, for a number of practical applications, it is not enough just to have lines with a high aspect ratio. It is also required that the lines have a low electrical resistivity ρ. Since the temperature of the heated substrate *T*_s_ in the process of aerosol jet printing, in addition to the aspect ratio, can also affect the spreading, drying, and sintering rates of the nano-ink, and as a result, the structure and morphology of the line and its electrical resistivity *ρ*. For this reason, the influence of the temperature of the heated substrate *T*_s_ in the range from 25 to 300 °C on the aspect ratio *AR*, resistivity *ρ*, morphology, and line structure was investigated.

**Figure 5:**
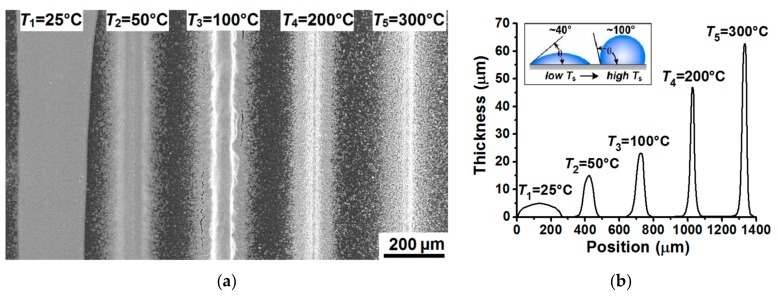
SEM image of lines formed by the method of aerosol jet printing on heated substrate, depending on the substrate temperature *T*_s_ equal to 25 °C, 50 °C, 100 °C, 200 °C, and 300 °C at *N* = 10 layers, *V*_s_ = 100 mm/min and *FR* = 2 (**a**); Relevant line cross-section profiles measured with an optical profilometer (**b**).

Figure 5a shows a SEM image of lines formed by aerosol jet printing on heated substrate, depending on the substrate temperature *T*_s_ equal to 25 °C, 50 °C, 100 °C, 200 °C, and 300 °C at *N* = 10 layers, *V*_s_ = 100 mm/min and *FR* = 2. In comparison with the previous experiment, the focusing ratio was reduced to 2 in order to demonstrate the effect of ink spreading on the substrate by increasing the amount of aerosol *Q*_a_ deposited on the substrate at *Q*_sh_ = const. The relevant cross-sectional profiles of the formed lines are shown in Figure 5b. The lines after printing were additionally sintered on a heated plate at a temperature of 400 °C for 60 minutes in order to ensure their conductive properties. The measured values of electrical resistivity *ρ*, aspect ratio *AR*, width *W*, thickness *t* and cross-sectional area of lines *S* depending on the temperature of the heated substrate *T*_s_ are presented in Table 2.

Figure 5a,b show that the temperature of the heated substrate *T*_s_ during aerosol jet printing has a significant effect on the geometry of the formed lines. Figure 5a shows that when aerosol jet printing of lines in the multi-pass mode at *N* = 10 layers, when the substrate temperature is *T*_s_ = 25 °C, a significant spreading of nano-ink is observed and, as a result, the lines are wide *W* ~ 250 μm and have low aspect ratio *AR* ~ 0.020, see Table 2. At the same time, it can also be seen from Figure 5b and Table 2 that even with a slight increase in the temperature of the heated substrate from 25 to 50 °C, a significant change in the geometry of the line is observed, namely, its width decreases significantly from ~ 250 to ~ 134 μm, and the thickness varies from ~ 5 to ~ 15 μm, which ultimately leads to an increase in the aspect ratio *AR* of the line from 0.02 to 0.11, respectively. This increase in the aspect ratio of the line is probably due to an increase in the contact angle *θ* due to a change in the composition of the nano-ink due to their evaporation upon deposition on a heated substrate. An increase in the contact wetting angle *θ* with an increase in the substrate temperature was previously studied in [41] in the process of capillary printing of chemical sensors. In work [41] It was found that the contact wetting angle *θ* can vary over a wide range from 30° to > 90° depending on the temperature *T*_s_ and the printing speed *V*_s_, and thus, to a large extent, determine the geometry of the formed lines from nano-ink. A schematic representation of the influence of the wetting angle *θ* on the geometry of the formed lines of nanomaterials with increasing temperature is shown in the inset in Figure 5b. The inset shows that, with an increase in the contact angle *θ*, a decrease in the width *W* and an increase in the thickness t of microdroplets on the surface are observed. In our experiments, as the substrate temperature increases from 100 to 300 °C, as in the work [41] due to the increase in the rate of evaporation of the nano-ink, the contact wetting angle *θ* is also likely to increase further, and as a result, there is a further increase in the aspect ratio of the lines from 0.17 to 0.70, respectively (see Figure 5b and Table 2).

It was also found that with increasing temperature of the heated substrate *T*_s_ in the range from 100 to 300 °C, the electrical resistivity of the formed lines increases from 2.56 ± 0.24 to 6.98 ± 0.73 μΩ∙cm, respectively (see Table 2). This increase in the electrical resistivity ρ can be explained by an increase in the porosity of the lines, due to the too rapid evaporation of the microdroplets of nano-ink at elevated temperatures in the range of 200–300 °C. An indirect confirmation of this statement is an increase in the cross-sectional area S of the lines from 1280 ± 225 to 2072 ± 352 μm^2^ with an increase in the substrate temperature from 100 to 300 °C, respectively (see Table 2). It is known that the amount of material deposited, which is proportional to the cross-sectional area of line *S*, should not depend on the substrate temperature *T*_s_. In this regard, it can be assumed that the increase in the cross-sectional area of the line *S* observed with increasing substrate temperature *T*_s_ is associated with an increase in the porosity of the formed lines. Thus, it was found that too high temperatures of the heated substrate *T*_s_ > 200 °C lead to the formation of lines with high values of electrical resistivity. 

In this regard, we can conclude that in the process of aerosol printing on a heated substrate in the multi-pass mode in order to form lines with a high aspect ratio and low resistivity, an optimal choice of substrate temperature is required. The optimum temperature of the substrate should contribute to a decrease in the spreading of the nano-ink and exclude the formation of a porous line structure, affecting their electrical and mechanical properties.

According to the results of adhesive tests, it was found that the adhesive strength of silver films formed on a heated silicon substrate at the optimum temperature *T*_s_ = 100 °C is 2.8 ± 0.9 N/mm^2^. This value of adhesive strength is sufficient for use in electronics with a high safety factor [42]. Thus, the suitability of the use of a heated substrate in the process of aerosol jet printing for the formation of conductive and adhesive-strong structures is further confirmed.

## 4. Conclusions

In this work, we studied the formation of silver lines on a heated silicon substrate during aerosol jet printing with varying the number of printing layers 1–10 and the temperature of the substrate 25–300 °C in order to obtain lines with a high aspect ratio > 0.1 and low electrical resistivity. As the deposited material, commercial silver nano-ink with a mass content of nanoparticles of about 46% was used. The temperature values responsible for the drying and sintering process of the nano-ink, which were 100 and 400 °C, respectively. It is established that the values of the aspect ratio of the line obtained during printing on a heated substrate at 100 °C are several times higher than under conditions of using an unheated substrate. It has also been found that the aspect ratio of the lines increases linearly with the number of print layers. In addition, it was found that with an increase in the temperature of the substrate from 25 to 100 °C, due to a decrease in the spreading of the nano-ink and an increase in the contact angle, there is a substantial increase in the aspect ratio of the formed lines from 0.02 to 0.17, respectively. Moreover, a further increase in the temperature of the substrate from 100 to 300 °C, in addition to increasing the aspect ratio of the lines from 0.17 to 0.70, due to an increase in the porosity of the structure, leads to an increase in the electrical resistivity from 2.56 to 6.98 μΩ∙cm, respectively. According to the results of adhesive tests, it was found that the approach of aerosol jet printing on a heated substrate is suitable for electronic applications, since the adhesive strength of silver films was 2.8 N/mm^2^. Thus, it has been experimentally shown that the formation of silver lines with a high aspect ratio > 0.1 and a low resistivity of 2-3 μΩ∙cm is advisable to be carried out at a substrate temperature of about 100 °C.

## Figures and Tables

**Table 1:** Parameters of the aerosol jet printing process to establish the effect of the number of printing layers *N* and the temperature of the heated substrate *T*_s_ on the characteristics of the lines formed.

Number of Printing Layers *N*	Substrate Temperature *T*_s_ (°C)	Printing Speed *V*_s_ (mm/min)	Focusing Ratio *FR*
1, 2, 5, 10	25, 100	100	5
10	25, 50, 100, 200, 300	100	2

**Table 2:** Parameters of the aerosol jet printing process to establish the effect of the number of print layers *N* and the temperature of the heated substrate *T*_s_ on the characteristics of the lines formed.

***T*_s_ (°C)**	25	50	100	200	300
***ρ* (µΩ∙cm)**	2.96 ± 0.28	2.69 ± 0.28	2.56 ± 0.24	3.53 ± 0.32	6.98 ± 0.73
***AR***	0.020 ± 0.004	0.11 ± 0.02	0.17 ± 0.03	0.47 ± 0.08	0.70 ± 0.12
***t* (μm)**	5.1 ± 0.9	15.2 ± 2.1	23.1 ± 3.2	46.9 ± 6.3	62.6 ± 8.4
***W* (μm)**	250.3 ± 23.6	134.2 ± 15.1	135.5 ± 14.6	99.1 ± 10.2	90.6 ± 9.4
***S* (μm^2^)**	1170 ± 234	970 ± 175	1280 ± 225	1462 ± 249	2072 ± 352
